# The *All of Us* dataset in scientific research: a bibliometric study of its utilization through 2024

**DOI:** 10.5195/jmla.2026.2346

**Published:** 2026-10-01

**Authors:** Yuqi He, Abrielle Spaletta

**Affiliations:** 1 yuqi.he@sjsu.edu, Engineering and Data Services Librarian, Dr. Martin Luther King Jr. Library, San José State University, San Jose, CA; 2 abrielle.spaletta@sjsu.edu, MLIS Student Research Assistant, San José State University, San Jose, CA

**Keywords:** *All of Us*, bibliometric analysis, publication trend, collaboration network, VOSviewer, Biblioshiny, dataset utilization, policy compliance

## Abstract

**Objective:**

This study presents a comprehensive bibliometric analysis of research publications utilizing the *All of Us* Research Program dataset to characterize publication trends, collaboration networks, key research topics, and compliance with *All of Us* publication and presentation policies through December 2024.

**Methods:**

A total of 561 publication titles were collected from the *All of Us* Publications Directory. Titles were then searched across Web of Science (WoS), Scopus, and PubMed, with WoS (475 publications identified) selected as the primary source for analysis. Productivity metrics, including publication growth, journal distribution, author productivity, and word frequency, were analyzed using Biblioshiny. Co-authorship and keyword co-occurrence networks were visualized in VOSviewer to reveal collaboration structures and emerging research themes.

**Results:**

Among the 475 analyzed publications, 2,803 authors were identified across 211 journals. The annual number of *All of Us* publications increased by 82.45% from 2015 to 2024. Major research themes included epidemiology, chronic conditions, genomics, and health disparities, with dermatology emerging as a particularly active area. Network analysis revealed strong intra-institutional ties and some cross-institutional collaborations, primarily among R1 universities.

**Conclusions:**

This bibliometric study demonstrates a rapid rise in scholarly activity leveraging the *All of Us* dataset, characterized by concentrated author and institutional engagement. These findings highlight both the growing impact of the *All of Us* dataset and opportunities to foster broader collaborations and diversify participation across the research community.

## INTRODUCTION

The *All of Us* Research Program [1], led by the National Institutes of Health (NIH) and launched nationally in May 2018, represents a pioneering initiative aimed at accelerating biomedical research and improving health outcomes for all. The program seeks to build one of the largest, most comprehensive, and most broadly accessible biomedical datasets in history, with a goal of enrolling over one million participants from diverse backgrounds in the United States, including underrepresented communities. As of July 2025, over 865,000 individuals have enrolled, with 745,000+ completing initial steps [2].

With the generous contributions of participants, the *All of Us* program has achieved enormous scale and impressive diversity. The program curates a comprehensive dataset that encompasses various health-related dimensions, including health questionnaires, electronic health records (EHRs), physical measurements, wearable devices, and genomics data [3]. Through the *All of Us* Research Hub (available at https://www.researchallofus.org/), researchers gain access to three tiers of data, ranging from aggregate data available to the public to individual-level data accessible via the Registered and Controlled Tiers [4]. The *All of Us* Researcher Workbench [5], launched in May 2020, is a collaborative platform that enables approved researchers in the Registered and Controlled Tiers to use cloud-based computing environments to analyze the *All of Us* data and pursue scientific inquiries.

Since the access to *All of Us* dataset expanded through the Researcher Workbench, scholarly output using the dataset has grown. Researchers are actively leveraging *All of Us* to explore a wide range of topics. As of July 2025, over 19,000 projects have been initiated through the Workbench [6] and hundreds of peer-reviewed articles have been produced [7]. This proliferation of research activity presents an opportunity to systematically assess how *All of Us* is influencing the scientific landscape.

Bibliometric analysis offers a powerful method for exploring this question. As a branch of Library and Information Science, bibliometrics analysis uses quantitative methods to analyze patterns in publication data. Common methodological approaches include publication counts to measure productivity, co-authorship network analysis to map collaboration patterns, citation analysis to measure impact, and co-word analysis to identify emerging themes. Bibliometric analysis has been used to evaluate researchers (e.g., [8]), institutions (e.g., [9]), research topics (e.g., [10]), theories (e.g., [11]), articles (e.g., [12]), books (e.g., [13]), journals (e.g., [14]), conferences (e.g., [15]), and even entire fields (e.g., [16]). These works demonstrate the flexibility of bibliometric analysis across scales—from single artifacts to entire disciplines.

Bibliometrics has also been applied to examine the impact of specific datasets in a few studies. Su et al. [17] conducted a bibliometric analysis to characterize the published papers utilizing the National Cancer Database (NCDB) data, aiming to understand the influence of the US NCDB dataset. Fan et al. [18] utilized co-word network modularity analysis (CWNA), a specific bibliometric method, to analyze journal articles citing the National Longitudinal Study of Adolescent to Adult Health (Add-Health) dataset from the ICPSR repository. Their study investigated major research topics associated with this dataset and their evolution over a two-decade span. Outside of health, Belter [19] measured the impact of three oceanographic datasets on the scientific community through citation counts. These examples illustrate how bibliometric approaches can trace the scholarly footprint of datasets and evaluate their role in advancing research.

Although *All of Us* has been evaluated on programmatic aspects, such as participant recruitment [20,21], data infrastructure [22,23], policy frameworks [24,25], and annual publication reviews have been conducted on its genomics subset [26], no studies have yet examined its overall scholarly impact. While these prior studies provide valuable information about the program’s design, goals and selected research outcomes, they do not examine how the broader research community is engaging with *All of Us* data or referencing the program in their scholarly work. A bibliometric perspective can fill this gap by mapping the evolution, pattern, and impact of *All of Us*-related publications.

This study presents the first bibliometric analysis of the *All of Us* Research Program’s scholarly output. Our goal is to characterize research utilizing the *All of Us* dataset, addressing the following four sets of questions:

### Publication Trends and Productivity

- What is the annual growth rate of publications?
- What are the predominant document types (e.g., journal articles, conference papers)?
- Which journals publish the most *All of Us*-related studies?
- Who are the most prolific authors contributing to this body of research?
- What institutions and countries are leading in research output?

### Collaboration and Network Analysis

- What are the patterns of collaboration among authors and institutions conducting *All of Us*-related research?

### Research Topics

- What are the primary research topics and themes emerging from studies utilizing the *All of Us* dataset?

### Policy Compliance

- To what extent do *All of Us*-related publications comply with the *All of Us* publication and presentation policy [27]?

By answering these questions, we aim to provide a comprehensive overview of how *All of Us* is shaping current and future directions in biomedical research. As the dataset is relatively new, citation analysis was excluded, as publications require at least two years to accrue meaningful citation counts [28]. Instead, we focus on publication trends, collaborative patterns, thematic content, and publication policy compliance to assess the early scholarly footprint of the *All of Us* Research Program.

## METHODS

### Data Collection

The *All of Us* Publications Directory page (available at https://www.researchallofus.org/publications/), accessed in April 2025) serves as a central repository for studies using the *All of Us* data. Since researchers using the Controlled and Registered Tiers must submit their publications for indexing [27], this resource was selected for data collection. An initial search was conducted on the *All of Us* publications page on March 8, 2024, resulting in 229 publications. To ensure a comprehensive dataset for annual analysis, the *All of Us* Publications page was monitored regularly, and the final search was updated on April 27, 2025 to include all studies published through December 31, 2024, resulting in a total of 561 publications. The flow chart in Figure 1 shows our data collection and analysis process.

**Figure 1:**
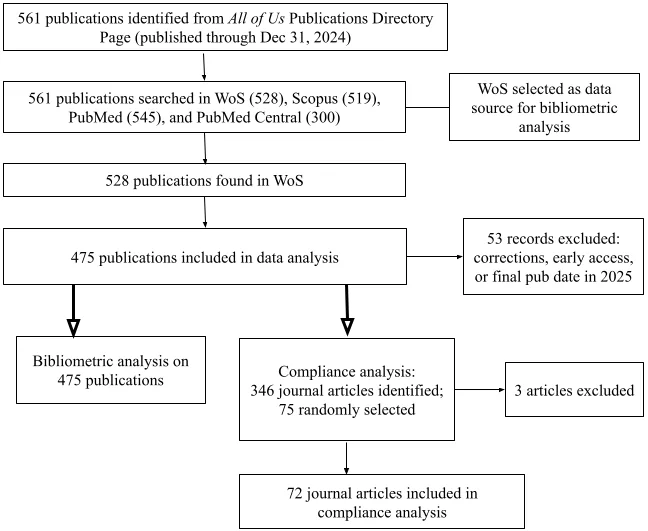
Data collection and analysis flowchart

Please note that the *All of Us* publications page is a dynamic website. As of our final data collection on April 27, 2025, it listed publications that include various document types including journal articles, conference proceedings, letters and more. However, when we revisited the site on May 19, 2025, it had been updated to display only peer-reviewed journal articles, with other document types moved to an expanded catalog [29]. A subsequent revisit on April 6, 2026, revealed substantial changes: the site adopted a new interface, added filters, reverted to including multiple document types, changed its URL to https://www.researchallofus.org/publication-directory/, and introduced AI-generated summaries for each publication. All our data analysis was based on the dataset collected from the *All of Us* Publication Directory page on April 27, 2025. Any updates or changes made to the website after this date, including additions, removals, or reclassifications of publication types, are not reflected in our analysis or findings.

Publication titles collected from the website were then searched across Web of Science Core Collection (SCI-Expanded, SSCI, AHCI, and ESCI), Scopus, PubMed, and PubMed Central. If no exact match was found, partial title searches were performed. A Data Log (available at https://osf.io/sxe6k/) created in Google Sheets was maintained to track searches across the four databases, documenting notes and search adjustments as needed. Of the 561 publications, 528 were found in Web of Science, 519 in Scopus, 545 in PubMed, and 300 in PubMed Central (see Figure 1). Given that Web of Science covers about 94% of the *All of Us* publications and is regarded as providing higher-quality bibliographic data than Scopus by the developers of our analysis tools, Biblioshiny and VOSviewer [30,31], it was selected as the primary data source for our bibliometric analysis.

To ensure to include only publications published through December 31, 2024, we applied additional filters in Web of Science, excluding document type: Correction and Early Access, and final publication year: 2025 before downloading the dataset. This filtering removed 53 records, resulting in a final dataset of 475 publications. To download the final dataset from Web of Science (WoS), the “Full Record” option was selected to include complete bibliographic information, and the dataset was saved as a plain text file.

### Data Analysis

For data processing and analysis, Google Sheets was used to record the dataset, track publications, generate basic counts, and create simple charts. The plain text file containing 475 publication records downloaded from WoS was then imported into specialized bibliometric visualization tools for further analysis. Biblioshiny [32], a web application for the R package Bibliometrix version 4.3.2, was primarily employed to calculate and visualize key productivity metrics, including the number of publications per year, journal distribution, and author productivity, as well as word clouds. To analyze collaboration patterns and research themes, VOSviewer [33] software version 1.6.20 was used for co-authorship network analysis and word co-occurrence visualization, providing insights into author and institutional collaborations and emerging research topics. In addition, OpenRefine [34], a data cleaning tool, was used to clean affiliations and author names before conducting network visualizations in VOSviewer.

To examine how *All of Us* publications adhere to the *All of Us* publication and presentation policy [27], we conducted a compliance analysis on a random sample of 75 articles drawn from the 364 peer-reviewed journal articles (filtered by Document Type: Article) in our 475-records WoS dataset. A full-dataset compliance review was not feasible due to the labor-intensive and manual nature of assessing each article against multiple policy criteria. We limited our analysis to peer-reviewed journal articles only, as they typically have higher word limits that allow for more comprehensive reporting and the *All of Us* publication and presentation policy [27] document outlines specific requirements applicable to this publication type. The *All of Us* policy has three key requirements. First, the policy mandates that authors submit the final, peer-reviewed manuscript to PubMed Central and make it publicly available immediately upon publication, without any embargo period. Second, it requires authors to acknowledge the contributions of *All of Us* participants and the program. Third, each article must include a statement on data availability or data access citing the Researcher Workbench and specifying the version of the *All of Us* dataset used.

Out of the 75 randomly selected journal articles, three were removed: one was an editorial letter, another was a journal article about program recruitment that did not use *All of Us* participant data, and one was a redacted journal article. This resulted in a final sample of 72 articles used for compliance analysis, approximately 20% of all peer-reviewed journal articles in our dataset. These 72 randomly-drawn articles provide a manageable yet representative sample for evaluating adherence to the *All of Us* publication and presentation policy. Full-texts of these 72 articles were then obtained. Author AS served as the primary coder for this compliance analysis. AS searched PubMed Central to verify article availability, reviewed each article's full text to evaluate the presence of appropriate acknowledgments and statements on data availability, and recorded the citation practices related to *All of Us* in the papers. Any articles marked as non-compliant in terms of acknowledgments or data statements were independently reviewed by author YH to validate the initial coding decisions. Discrepancies were resolved through discussion to reach consensus.

## RESULTS

### Publication Trends and Productivity

#### The Number of Publications

Of the 475 publications examined in this study, the majority (245 publications, 52%) were published in 2024. From 2015 to 2024, there was an overall growth rate of 82.45% in the number of papers published annually, indicating a rapid increase in scholarly activity utilizing the *All of Us* dataset. This trend is illustrated in Figure 2. A small number of publications appeared prior to the program’s national launch in 2018 and these early works primarily focused on the planning of the initiative. Beginning in 2021, the number of publications using *All of Us* data began to grow exponentially, continuing this upward trajectory through 2024. This is largely due to the launch of the Researcher Workbench in May 2020, enabling broad access to the *All of Us* dataset for the scientific community.

**Figure 2:**
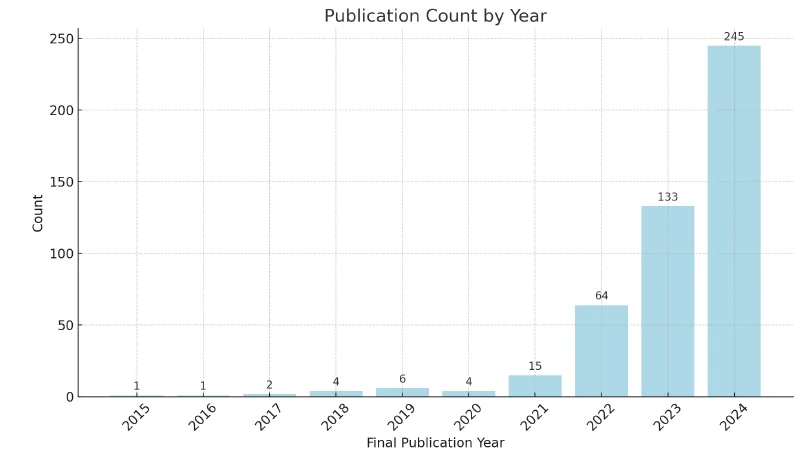
Publication count by year

#### Document Types

Figure 3 shows the breakdown of document types across the 475 publications based on the WoS Core Collection document types categories. Journal articles (69.9%) make up the majority of published items, followed by Letters (19.5%), Editorial Materials (5.1%), Meeting Abstracts (2.5%), Review Articles (2.5%), and Proceeding Papers (0.6%).

**Figure 3:**
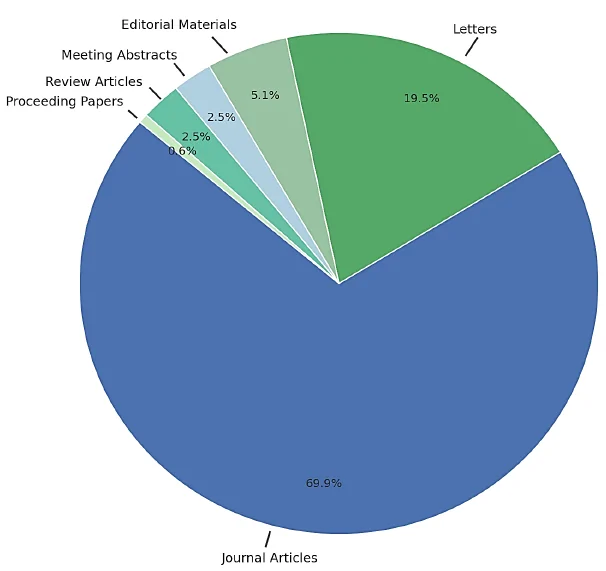
Document types

#### Journals

From 2015 to 2024, a total of 211 journals published papers utilizing the *All of Us* dataset. Table 1 presents the top ten journals in terms of the number of *All of Us* related research published. The *Journal of the American Medical Informatics Association* (JAMIA) ranks first, reflecting its strong alignment with the informatics-driven nature of the *All of Us* initiative. Both the second- and third-ranked journals specialize in dermatology. In fact, five of the top ten journals are dermatology-focused, suggesting a particularly high level of engagement with the *All of Us* dataset within the dermatology research community.

**Table 1:** Top journals publishing *All of Us* research

Publication Titles	Record Count	% of 475
Journal of the American Medical Informatics Association	49	10.32%
Archives of Dermatological Research	34	7.16%
Journal of the American Academy of Dermatology (JAAD)	31	6.53%
Plos One	14	2.95%
Nature Communications	11	2.32%
International Journal of Dermatology	9	1.89%
Clinical and Experimental Dermatology	8	1.68%
JAMA Network Open	8	1.68%
Nature Genetics	7	1.47%
JAAD International	6	1.26%

#### Authors

A total of 2803 authors utilized the *All of Us* dataset. Figure 4 shows the most prolific authors, with Cohen JM leading with 42 publications, followed by Leasure AC with 23 publications, and Baxter SL with 21 publications. In particular, the top two authors are from the same institutions and frequently co-author with each other, with a primary focus on dermatology. The third author Baxter SL’s research focuses on ophthalmology, particularly glaucoma. When examining the publication timelines of the top 10 authors (see Figure 5), five began publishing in 2021, shortly after the Researcher Workbench became available in May 2020, suggesting they were early adopters of the *All of Us* dataset. All top 10 authors have consistently published *All of Us*-related research for at least three years after initially utilizing the dataset.

**Figure 4:**
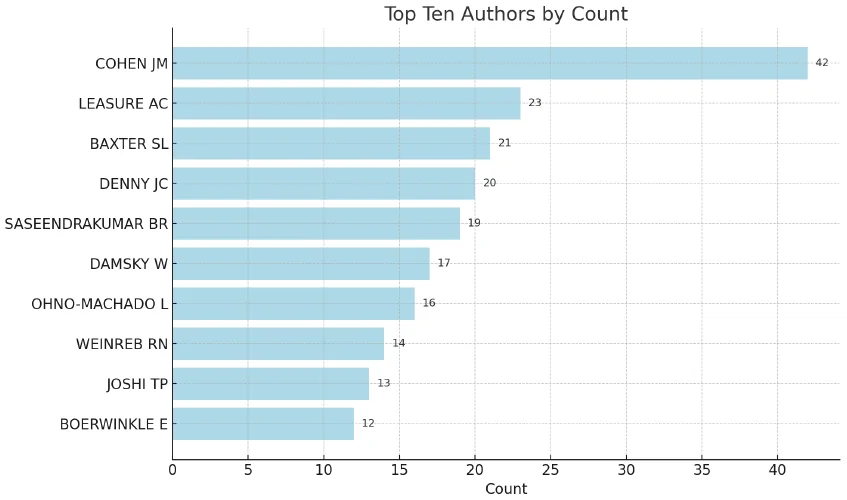
The most prolific authors

**Figure 5:**
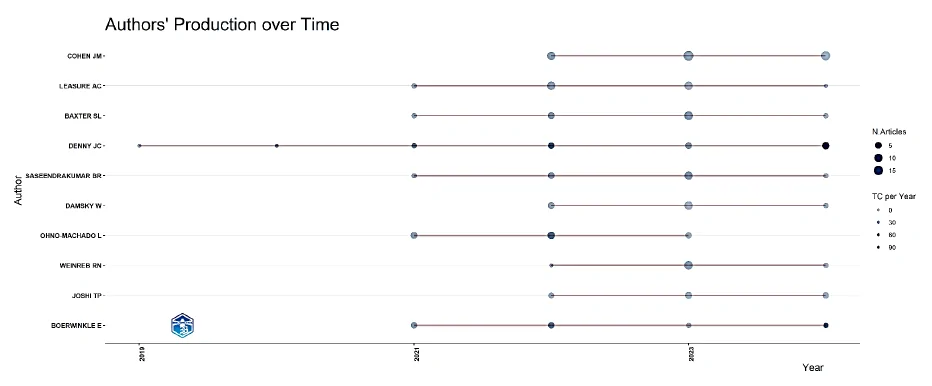
Top 10 authors’ production over time

#### Institutions

Research utilizing the *All of Us* dataset has been conducted across 520 institutions, with 20% (99) of those institutions contributing at least three publications to the literature. Figure 6 highlights the top ten institutions based on publication count, all of which are located within the United States. The publication counts were calculated using author affiliations, with each institution receiving one count per article, regardless of the number of affiliated co-authors. Universities with a strong focus on medical research, such as Vanderbilt, UC San Diego, Johns Hopkins, are among the top users of this dataset. Major health institutions and hospitals, like the NIH and Massachusetts General Hospital, also play a leading role in utilizing the *All of Us* dataset.

**Figure 6:**
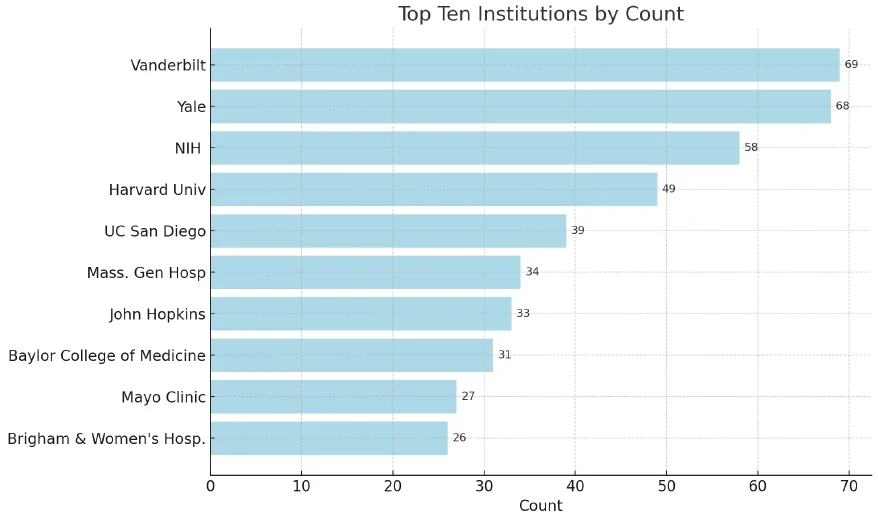
Top 10 institutions

#### Countries

Papers utilizing the *All of Us* dataset were published primarily by authors based in the United States, with a low international co-authorship of 12.87%. This limited global collaboration is not surprising, as the *All of Us* Research Program collects data exclusively from individuals living in the US, and institutions that have signed the Data User and Registration Agreement (DURA) are predominantly US-based.

### Collaboration and Network Analysis

#### Individual Co-authorship Map

The co-authorship collaboration map (see Figure 7) shows the names of authors who have published at least three papers using the *All of Us* dataset. The size of each circle reflects the number of papers published by an author, while the lines connecting circles indicate co-authorship. Colors represent clusters of closely connected authors, who often belong to the same institution. The network map reveals that while many authors collaborate primarily within their institutions, cross-institutional collaboration is also common. A few authors have published independently without co-authors. In addition, authors with larger circles such as Cohen, Leasure, and Baxter stand out. This is consistent with Figure 4 on the top authors.

**Figure 7:**
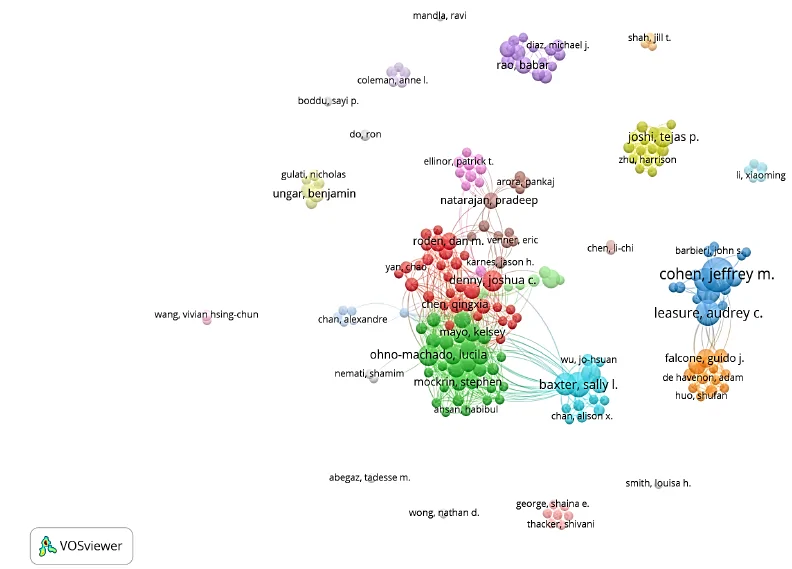
Individual co-authorship network

VOSviewer parameters: full counting, a minimal of 3 publications, ignore papers with more than 25 authors, show all items. Interactive map: https://tinyurl.com/27fzlbud.

#### Institutional Co-authorship Map

The institutional co-authorship map (see Figure 8) displays institutions with at least five *All of Us* publications and at least one cross-institutional collaboration. A total of 57 institutions are represented in the graph. The network map shows collaborations among them. A thesaurus file was uploaded to VOSviewer to consolidate institution names, particularly those with medical campuses or specific departments cited. In the map, larger circles indicate higher publication counts. Leading institutions, such as Vanderbilt, Yale, the NIH, Harvard, and UC San Diego, stand out prominently. This is consistent with Figure 6 on the top institutions.

**Figure 8:**
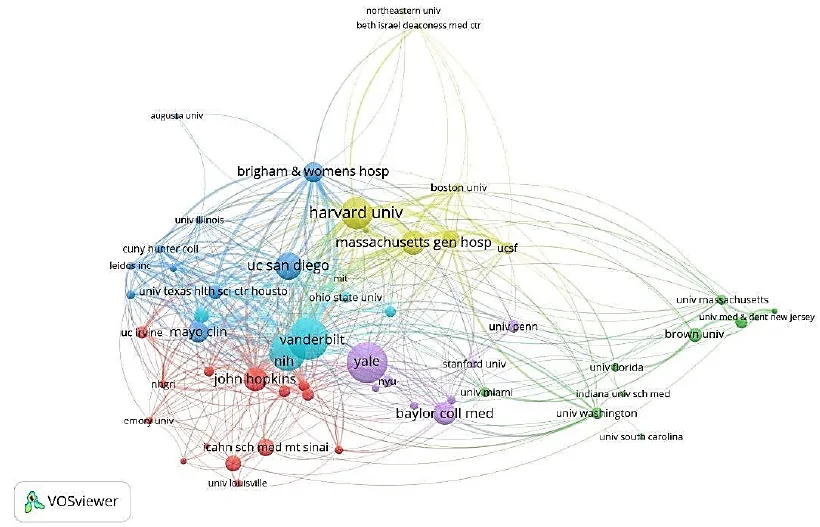
Institutional co-authorship network

VOSviewer parameters: full counting, a minimal of 5 publications, ignore papers with more than 25 authors, only show connected items. Interactive map: https://tinyurl.com/23g35buf.

### Research Topics

#### WOS Categories

Table 2 listed the top ten Web of Science categories for our dataset. These categories are assigned at the journal level and a single journal can be assigned to multiple WoS categories. Dermatology is the most prolific category listed at 115 records, which is nearly double the number of records of Health Care Sciences Services, the second largest category at 67 records.

**Table 2:** Web of Science categories

Web of Science Category	Record Count	% of 475
Dermatology	115	24.21%
Health Care Sciences Services	67	14.10%
Medical Informatics	64	13.47%
Computer Science Interdisciplinary Applications	55	11.57%
Computer Science Information Systems	51	10.74%
Information Science Library Science	49	10.32%
Medicine General Internal	40	8.42%
Multidisciplinary Sciences	37	7.79%
Genetics Heredity	34	7.16%
Ophthalmology	29	6.11%

#### Word Co-occurrence Maps

**Keyword Map**

The keyword map (see Figure 9) is based on the titles and abstracts of the publications in our dataset. Keywords were extracted using full counting, with a minimum threshold of eight occurrences per term, resulting in six clusters. This visualization highlights frequently mentioned terms with larger boxes, while terms that are spatially closer and share the same color indicate high levels of co-occurrence within the dataset. The blue cluster represents research centered on genomics and precision medicine. The red cluster focuses on chronic diseases (especially dermatological and metabolic) and their study within diverse or underrepresented populations. The bright greenish-blue cluster reflects studies of mental health conditions, especially depression and anxiety. The green cluster centers on barriers and facilitators in healthcare access, provider interactions, and health equity. The yellow cluster centers on the *All of Us* program, infrastructure and community. Finally, the small purple cluster reflects computational methods such as machine learning algorithms used to analyze the dataset.

**Figure 9:**
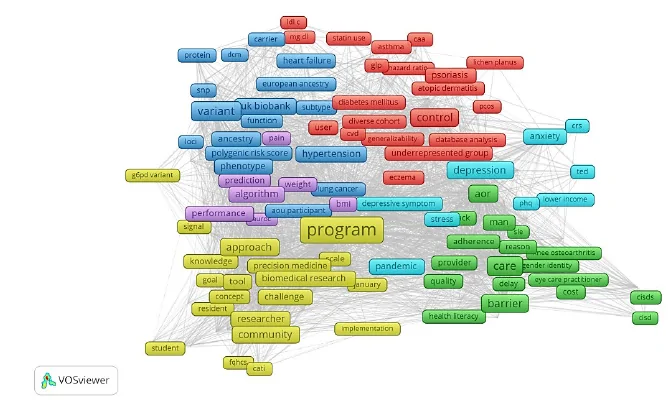
Keyword Map

VOSviewer parameters: full counting, a minimal of 8 occurrences per term, association strength normalization, 0.75 resolution for cluster analysis, box-style labeling was chosen for better visibility. Interactive map: https://tinyurl.com/29bs55be.

**MeSH Term Maps**

The MeSH term map (see Figure 10), created using MeSH terms downloaded from PubMed, visualizes the co-occurrence patterns of MeSH terms assigned to *All of Us*-related research. Only terms with a minimum of five occurrences were included, and cluster analysis resulted three main clusters. Unlike the box-style labeling used in Figure 9, Figure 10 employs the traditional circle label format, where the size of each circle reflects the frequency of term occurrence. Featured at the center of the map are terms such as “humans,” “adult,” “male,” “female,” “United States”, and “population health,” underscoring *All of Us*’ central focus on human subjects in the United States and population-based research. The thematic clusters revealed by MeSH term co-occurrence closely align with those observed in the title and abstract term maps. The red cluster highlights *All of Us*’ contributions to precision health and genomics research, often supported by electronic health records and advanced computational methods such as machine learning. The green cluster centers on population health, social determinants of health, and health equity, frequently investigated using survey data and sociodemographic variables provided by the *All of Us* dataset. Lastly, the blue cluster reflects disease-specific research and related methodological approaches, for example dermatology and mental health, and their study designs like case-control studies.

**Figure 10:**
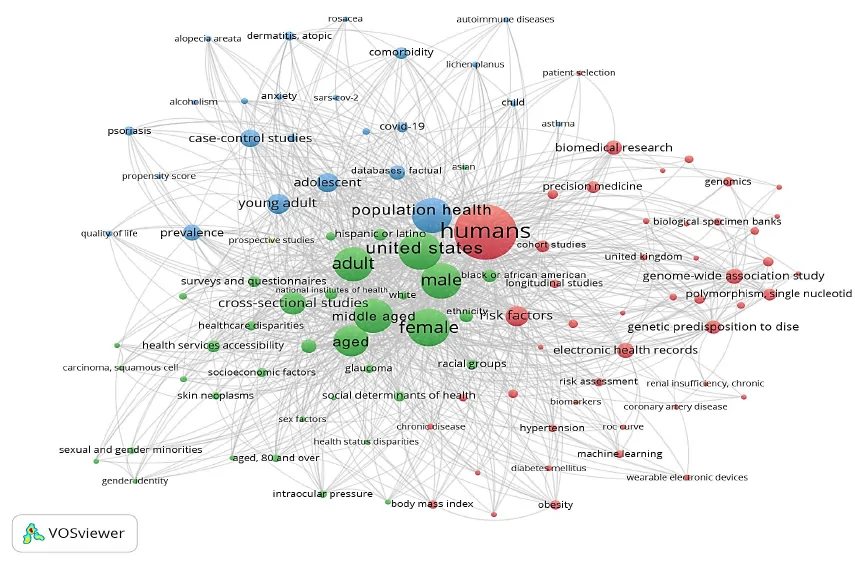
MeSH Term Map

VOSviewer parameters: full counting, a minimal of 5 occurrences per term, association strength normalization, 0.75 resolution for cluster analysis, light-gray connected lines were chosen for better visibility. Interactive map: https://tinyurl.com/2xu7lqr4.

**Word Clouds**

To further explore the thematic focus of *All of Us*-related research, two word clouds were generated using Biblioshiny to visualize the most frequently used terms. These visualizations offer an alternative perspective on keyword usage patterns across the dataset. Figure 11 displays the top 50 terms from Keyword Plus on the WoS platform, which are algorithmically generated terms based on cited references and provide insight into broader contextual themes. Figure 12 presents the top 50 author keywords, which reflect the authors’ intentional framing of their research topics. Together, these word clouds highlight recurring research topic themes such as epidemiology, health disparities, chronic conditions (e.g., diabetes, hypertension, mental health), precision medicine, and the use of large-scale health data. While there is overlap between the two sets of keywords, differences also emerge: author keywords tend to more specific, such as “*All of Us*” or “*All of Us* Research Program”, reflecting the absence of an established subject heading for this dataset, whereas Keyword Plus terms offer a broader thematic scope. These patterns reinforce findings from the abstract/title and MeSH term analyses, illustrating the multidisciplinary and data-driven nature of *All of Us* research.

**Figure 11:**
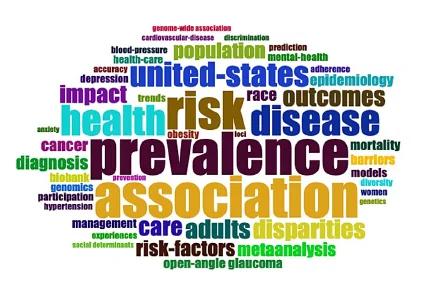
Word cloud on Keyword Plus (50 words)

**Figure 12:**
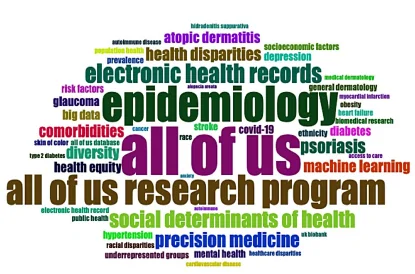
Word cloud on author’s keywords (50 words)

### Compliance

Our compliance analysis evaluated how well *All of Us* journal articles adhere to three key requirements of the *All of Us* publication and presentation policy [27]: availability in PubMed Central, inclusion of an acknowledgment of *All of Us* program and participants, and inclusion of a statement on data availability or access

In our random sample of 72 journal articles, only 55% (40/72) found free full-text access in PubMed Central. Among the 32 articles not available in PubMed Central, 8 were under embargo at the time of analysis. Regarding acknowledgement, 73% (52/72) of the sampled articles included a statement recognizing *All of Us* participants and the program. A similar proportion, 71% (51/72) included a statement on data availability or data access. Overall, 58% of the publications included both an acknowledgment and a statement on data availability.

Among the 52 articles that included an acknowledgment of *All of Us* participants and the program, we also examined where these statements were located. The majority (79%, 41/52) placed the acknowledgment in a stand-alone Acknowledgments section, typically located at the end of the paper, just before the references. Seven articles included the acknowledgment within the funding section, while four placed it in other sections such as contributors/contributions, the appendix, or footnotes.

The location of the statement on data availability or data access was more varied. Approximately 47% (24/51) placed the statement in a dedicated Data Availability Statement section, while 39% (20/51) included it within the Methods section. The remaining seven articles included the statement in other locations, such as the Introduction.

We further analyzed how the *All of Us* program is cited in research publications by categorizing citations into three types:

- Formal Citation: The paper references at least one publication specifically about the *All of Us* program and includes it in the reference list.
- Informal Citation: The paper mentions and discusses the *All of Us* program but does not cite a specific publication in the reference list.
- No Citation: The paper does not mention or cite the *All of Us* program in any form.

In our 72-article dataset, most papers (61/72) included a formal citation, informal citations appeared occasionally (10/72), and only one paper lacked any citation. Table 3 lists the most frequently cited *All of Us* program publications (excluding citations to the Researcher workbench), noting that one article might cite multiple *All of Us* program publications.

**Table 3:** Most frequently cited publications about the *All of Us* Program

Full Citation	Number of Documents (n=61)
The *All of Us* Research Program Investigators. The “*All of Us*” Research Program. N Engl J Med. 2019 Aug 15;381(7):668–76.	57
Mapes BM, Foster CS, Kusnoor SV, Epelbaum MI, AuYoung M, Jenkins G, et al. Diversity and inclusion for the *All of Us* research program: A scoping review. PLOS ONE. 2020 July 1;15(7):e0234962.	10
Ramirez AH, Sulieman L, Schlueter DJ, Halvorson A, Qian J, Ratsimbazafy F, et al. The *All of Us* Research Program: Data quality, utility, and diversity. Patterns. 2022 Aug 12;3(8):100570.	7
Ramirez AH, Gebo KA, Harris PA. Progress With the *All of Us* Research Program: Opening Access for Researchers. JAMA. 2021 June 22;325(24):2441–2.	3
Sankar PL, Parker LS. The Precision Medicine Initiative’s *All of Us* Research Program: an agenda for research on its ethical, legal, and social issues. Genetics in Medicine. 2017 July 1;19(7):743–50.	2
Collins FS, Varmus H. A New Initiative on Precision Medicine. N Engl J Med. 2015 Feb 26;372(9):793–5.	2

## DISCUSSION

### *All of Us* Utilization

The findings from this bibliometric analysis reveal several early trends in how the *All of Us* dataset is being utilized in the research community. Our analysis demonstrates a rapid increase in scholarly activity following the launch of the Researcher Workbench in 2020, concentrated author and institutional contributions, and a strong but uneven thematic focus. These patterns suggest both areas of high engagement with the *All of Us* resource as well as notable gaps that merit attention.

The sharp increase in publication volume, particularly between 2022 and 2024, illustrates a growing adoption of the *All of Us* dataset. Over half of the 475 analyzed publications were published in 2024 alone. This rapid growth underscores the success of the *All of Us* Research Workbench in enabling researcher access and reflects a rising interest in large-scale, diverse biomedical datasets in the research community. However, publication productivity is heavily concentrated among a small number of authors at a few elite research universities or health institutions: the top five authors contributed nearly 20% of all publications, and the top five institutions produced over half. While these contributors likely benefit from strong research infrastructure and support for health science in general, their outsized presence points to *All of Us*’s early adoption by a relatively narrow segment of the research community. The dominance of R1 universities with affiliated medical centers in both publication output and collaboration networks further emphasizes this concentration. In contrast, smaller institutions, teaching-focused universities and minority-serving institutions (MSIs) are largely absent from the top tier of contributors. This under-utilization may reflect barriers such as limited funding, lack of infrastructure, or insufficient awareness of *All of Us* resources. Addressing these gaps could broaden participation and enhance the diversity of perspectives in *All of Us*-based research.

Co-authorship and institutional network maps reveal moderately dense collaboration patterns, particularly among top authors and leading institutions. These patterns are characterized by strong intra-institutional ties and some cross-institutional collaboration. The collaboration maps further highlight that a small group of authors and institutions drive much of the early scholarly activity utilizing the *All of Us* dataset. It is also worth noting that when examining the top 10 authors’ publication productivity over time (see Figure 5), a clear trend emerges: once researchers begin working with the *All of Us* dataset, they tend to continue publishing *All of Us*-related studies year after year. This sustained engagement suggests that *All of Us* serves not only as an initial research opportunity but also as an ongoing resource that supports long-term scholarly productivity. This is likely due to the dataset’s longitudinal design, regularly updated data, and accessibility through the Workbench. This sustained engagement could serve as a compelling marketing point for attracting new researchers, highlighting the *All of Us* dataset not only as a valuable resource for initial studies but also as a platform that supports ongoing, long-term research agendas.

Word co-occurrence analysis of titles, abstracts, and MeSH terms reveals a strong focus on dermatology, genomics, and chronic conditions such as diabetes and mental health. Dermatology, in particular, appears to be an area of concentrated engagement, dominating both publication volume and author representation. Five of the top ten journals publishing *All of Us* research are dermatology-focused, and the most prolific authors share this research specialty. While this reflects a valuable application of the dataset within specific health domains, it also suggests that *All of Us*’s broader multidisciplinary potential remains underutilized. The dataset holds substantial potential for a wide range of research areas, and as more researchers gain access and familiarity with its contents, new lines of inquiry are likely to emerge.

### Compliance with *All of Us* policy

In addition to conducting a bibliometric analysis of publications utilizing the *All of Us* dataset, our research also investigates authors’ degree of compliance with *All of Us* publishing policies. These policies require authors to submit a final, embargo-free manuscript of their peer-reviewed work to PubMed Central, acknowledge the *All of Us* program and participants, and include a statement on data availability or access.

In our sample of 72 journal articles, only about 55% of publications were available in PubMed Central, suggesting that many papers are inaccessible. After accounting for 8 articles that were under embargo and noting that *All of Us* has appended a note to their reporting form clarifying that the embargo-free clause is currently not enforced [35], 24 articles remain (33% of the sample) not accessible in PubMed Central. This suggests that these authors did not submit their manuscripts to PubMed Central as required. Based on our analysis, we estimate that approximately one-third of *All of Us* publications are not deposited in PubMed Central, raising concerns about overall compliance with *All of Us*’s public access policy.

58% of our sample papers provide both an acknowledgement statement and a data availability statement, which reveals a wide gap in policy compliance. Though authors are required to include these statements and self-report their own compliance to the *All of Us* program upon journal submission, our results show that many are not including the required information, or they include only one type of statement. Partial compliance, or lack thereof, implies that there is divergence from *All of Us* policy expectations and actual publishing practices.

### Implications for Libraries

Based on these findings, there are a few implications for libraries. Libraries can play a role in promoting equitable access to and use of the *All of Us* dataset. The concentration of *All of Us*-related research at a small number of elite institutions points to potential gaps in awareness, capacity, or infrastructure at smaller, under-resourced institutions. This suggests an opportunity for libraries, particularly those at minority-serving institutions (MSIs) or teaching-focused universities, to participate in outreach and education efforts by raising awareness of the *All of Us* Researcher Workbench and its potential for inclusive health research on their campuses. Simple approaches might include adding *All of Us* into relevant LibGuides or introducing the dataset to faculty and students as needs arise. Additionally, libraries with more developed data services can provide onboarding support, training, and sustained guidance for new users, helping researchers and students navigate the platform and integrate it into their work. Through these efforts, libraries can help broaden participation and support a more diverse range of voices and institutions represented in *All of Us*-related research.

### Limitation

There are several limitations to this bibliometric study. First, bibliometric analyses are inherently dynamic, as publication trends and citation patterns evolve over time. The results of this study could change significantly if the utilization of the *All of Us* dataset were examined in the future. Second, since the publications included in this study are relatively recent, we did not analyze citation impact. Citation-based bibliometric indicators typically require articles to be at least two years old to accumulate a sufficient number of citations for reliable assessment [28]. Thus, our study does not evaluate citation impact of research utilizing the *All of Us* dataset, missing an important component in measuring its influence. Finally, our analysis only included publications that used the *All of Us* dataset through registered and controlled tier access. Studies utilizing data from the public tier, which does not require registration, were not captured in our data collection method. This limitation suggests that our findings may underestimate the overall impact of the dataset in the scientific community. Future research could employ alternative collection methods, such as tracking publications across broader sources or analyzing social media engagement, to provide a more comprehensive understanding of the dataset’s utilization and influence.

## Data Availability

Data associated with this article are available in the Open Science Framework at https://osf.io/sxe6k/.

## SUPPLEMENTAL FILES

**Appendix A:** Data Log
